# Methodology for Fast and Facile Characterisation of Carbon-Based Electrodes Focused on Bioelectrochemical Systems Development and Scale Up

**DOI:** 10.3390/ma10010079

**Published:** 2017-01-20

**Authors:** Raúl Mateos, Raúl M. Alonso, Adrián Escapa, Antonio Morán

**Affiliations:** 1 Chemical and Environmental Bioprocess Engineering Group, Natural Resources Institute (IRENA), Universidad de León, Av. de Portugal 41, 24009 León, Spain; rmatg@unileon.es (R.M.); ralog@unileon.es (R.M.A.); amorp@unileon.es (A.M.); 2 Department of Electrical Engineering and Automatic Systems, Universidad de León, Campus de Vegazana s/n, 24071 León, Spain

**Keywords:** bioelectrochemical systems, carbon-based electrodes, electroactive area, fractal dimension, scale up

## Abstract

The development and practical implementation of bioelectrochemical systems (BES) requires an in-depth characterisation of their components. The electrodes, which are critical elements, are usually built from carbon-based materials due to their high specific surface area, biocompatibility and chemical stability. In this study, a simple methodology to electrochemically characterise carbon-based electrodes has been developed, derived from conventional electrochemical analyses. Combined with classical electrochemical theory and the more innovative fractal geometry approach, our method is aimed at comparing and characterising the performance of carbon electrodes through the determination of the electroactive surface and its fractal dimension. Overall, this methodology provides a quick and easy method for the screening of suitable electrode materials to be implemented in BES.

## 1. Introduction

Bioelectrochemical systems (BES)—an innovative technology in the fields of electrochemistry and bioprocessing technologies [1]—have undergone rapid development, breaking through as promising alternatives in the fields of wastewater treatment [2], bioremediation [3], biosensors construction [4] and chemicals recovery [5].

For wastewater treatment and chemicals recovery applications in particular, BES have reached a degree of maturity that has allowed researchers and engineers to bring about the first pilot-scale experiments [6,7]. These experiments provide valuable information on chemical, process engineering or durability issues (among many others), all of which helps to pave the way to practical implementation [8,9]. However, to optimize the reactor’s performance, BES developers often have to face the challenge of selecting the most appropriate electrode materials, since they play a vital role on biolectrochemical reaction rates or energy loses. This is not always a straightforward issue since there are a wide variety of potential electrode materials [10]. Thus, when developing new BES, having a fast and easy method for the screening and characterization of electrode materials could become a powerful tool that can result in substantial time and resources savings.

The presence of surface patterns on electrodes is a key aspect when selecting electrode materials for BES, as it has a significant effect on their electrochemical performance [11]. The electroactive area (EA) of an electrode is a parameter clearly related to its surface structure, and its determination represents an essential step in characterising the electrochemical behaviour of electrodes in electrochemical systems in general [12] and BES in particular [13]. By combining EA determination with other analyses such as stochastic geometrical pattern characterisation, the three-dimensional structure of a porous electrode and its performance can be accurately estimated. Some studies make use of complex numerical treatments focused on a specific porous electrode type, which provide accurate results [14,15]. Still, these approaches require the development of “tailor-made” analysis strategies for each individual porous electrode, which usually results in time-consuming analysis methods, and complicates comparison between electrodes.

In this paper, we present an easy and simple method for a preliminary characterisation of electrode materials for BES. It is based on conventional electrochemical techniques and allows for fast and reliable estimation of the active area and electrode surface configuration of electrode materials. The method here proposed is intended to provide researchers and engineers with a tool for a rapid and easy characterization of potentially suitable electrode materials for BES applications.

## 2. Experimental

### 2.1. Methodology Proposal

The core of this methodology relies on the determination of two basic parameters: the electroactive area (EA) and fractal dimension (*D_f_*) whose calculations are detailed in Section 2.2 and Section 2.3 respectively. While the EA provides a fairly good approximation of the equivalent surface area of a flat electrode (which is related to electrochemical reaction rates), the fractal dimension highlights the presence of three-dimensional patterns on the surface of the electrode (electrodes with three-dimensional structure tend to facilitate the settling and proliferation of electroactive microorganisms). Therefore, the information provided by these two parameters is complementary, and can be combined for preselecting the most suitable electrode material for a particular BES design.

The main advantage of this method, aside from its simplicity and promptness, is that it only requires performing basic electrochemical analytical techniques which are available on every electrochemical laboratory. These techniques are described in the supporting information.

### 2.2. Determination of Electroactive Area

A direct method for the determination of the electroactive area is evaluation of the peak current in a set of cyclic voltammetry (CV) experiments using a well-known redox couple and cell set-up [16] (in this study K_3_Fe(CN)_6_/K_4_Fe(CN)_6_), that usually requires ohmic drop compensation to obtain suitable data for further analysis [17]. In our particular case, the ohmic drop is calculated by averaging the results obtained from current interrupt (CI) and electrochemical impedance spectroscopy (EIS) (see supporting information, Figures S1 and S2).

The EA can be determined from Equation (1) for a Nerstian system. Peak current (*I_p_*) can be calculated in a CV according to the Randles-Ševčik equation:

(1) $$I_{p}=0.4463{\left(\frac{F^{3}}{RT}\right)}^{1/2}\;A\;C\;n^{3/2}\;{\left(D\;\upsilon \right)}^{1/2}$$

where *I_p_* is the peak current in A, *F* is the Faraday’s constant in C·mol^−1^, *R* is the ideal gas constant in J·K^−1^·mol^−1^, *T* is the absolute temperature in K (298 K in this study), *A* is the electroactive area in cm^2^, *D* is the diffusion coefficient of the electroactive specie in cm^2^·s^−1^, *n* is the number of electron transferred in the redox reaction, *C* is the bulk concentration of the electroactive compound in solution in mol·cm^−3^, and *υ* is the scan rate in V·s^−1^ [18]. The value of the diffusion coefficient is 0.76 × 10^5^ cm^2^·s^−1^ at the experimental temperature of 25 °C in KCl 0.1 M and it was obtained from bibliographic data [19].

Cathodic peak currents (*I_pc_*) can be obtained from CV experiments using the decaying anodic current as baseline [18]. From the slope of the linear fit between *I_pc_* and the square root of the scan rate, the electroactive area can be determined, following Equation (1). This approach can only be applied in the experimental range in which a linear trend is observed between *I_pc_* and *υ*^1/2^. The described approach is used for comparative purposes, keeping in mind that this model applies for flat electrodes [18].

Although more accurate numeric treatments have been developed [14], this study implements a simple model for the wide range of materials tested.

### 2.3. Determination of Fractal Dimension (D_f_)

Roughness is a key parameter in electrode behaviour because it can condition mass transfer [20] and biofilm development [21]. Our method relies on the use of a fractal geometry approach to characterise electrode surface properties related to self-similarity.

Since Mandelbrot carried out his work on fractal geometry [22], it has been used to model different systems in science and technology [23], and especially in electrochemistry, due to the importance of electrode surface characteristics [14,15,24]. *D_f_* is a quantitative parameter that can be used to analyse the rough surface structures of an electrode [15,17,24].

A method for determining *D_f_* from CV data was proposed in [14,25]. This method consists of estimating the value of the fractal parameter (α) from the peak current of a set of voltammograms considering that:

(2) $$I_{pc}\;\propto \;\upsilon^{\alpha }$$

As a consequence, by plotting the peak current vs. ʋ on a logarithmic scale, the fractal parameter can be estimated from the slope of the fitted linear model. Ohmic losses must be negligible in order to apply this methodology.

The fractal parameter is related to the fractal dimension through:

(3) $$D_{f}=2\alpha +1$$

*D_f_* values higher than 2 imply rough three-dimensional electrode surfaces whose macroscopic areas are lower than their microscopic areas [26]. In the case of a flat electrode, the *D_f_* value is expected to be 2, corresponding to a fractal parameter of 0.5. Lower values of *D_f_* can be attributed to inactive surface regions that lower the electroactive surface area below the equivalent flat area.

An uncertainty estimation for *D_f_* can be provided via the confidence intervals of the slope parameter obtained from the fitted linear model, once the normality of the residuals has been checked.

### 2.4. Method Validation

**Figure 1:**
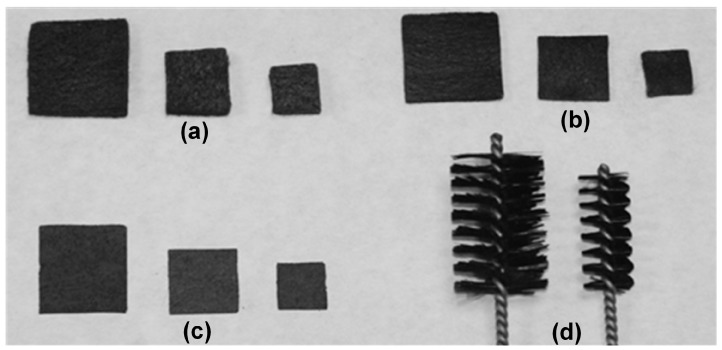
0.5 cm-thick carbon felt (**a**); 0.25 cm-thick carbon felt (**b**); carbon paper (**c**); and brush (**d**) electrodes.

The characterisation method described above was validated on four different types of carbon-based materials: Carbon felt of two different thicknesses (SGL Group), carbon paper (SGL Group) and carbon brush (Millrose Co., Mentor, OH, USA), shown in Figure 1. All of these materials were tested in different widths and lengths. The materials are specified in Table 1. See supporting information (Section 3; Figure S4) for apparent surface determination of carbon brush.

**Figure 2:**
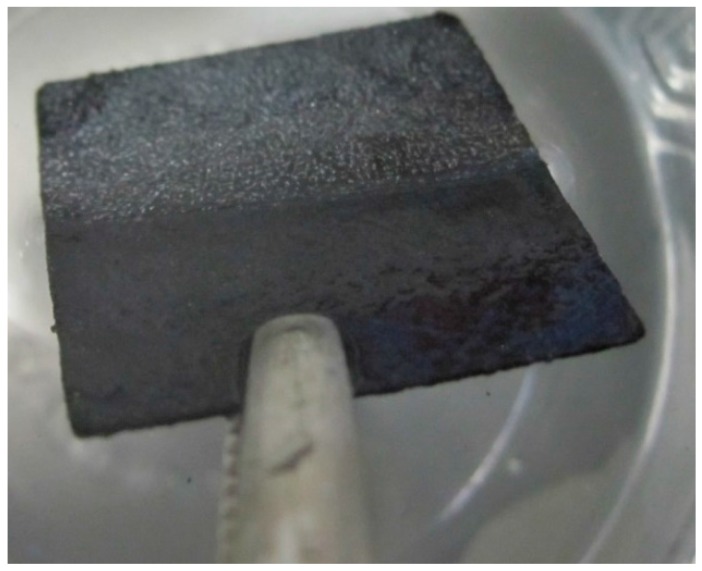
Carbon paper electrode with air bubbles after pre-treatment.

Most of these materials present unacceptable initial wettability that may distort the analytical results, meaning that a pre-treatment to mitigate this problem is necessary [27,28]. The pre-treatment consists of sequentially immersing the electrode into 1 M nitric acid, acetone and ethanol solutions with concentrations for 12 h, 30 min and 30 min, respectively [27]. These parameters were established based on previous experiments carried out on carbon felt materials, but proved to be ineffective for air removal from carbon paper electrodes, as shown in Figure 2.

### 2.5. Cell Set-Up and Instrumentation

**Figure 3:**
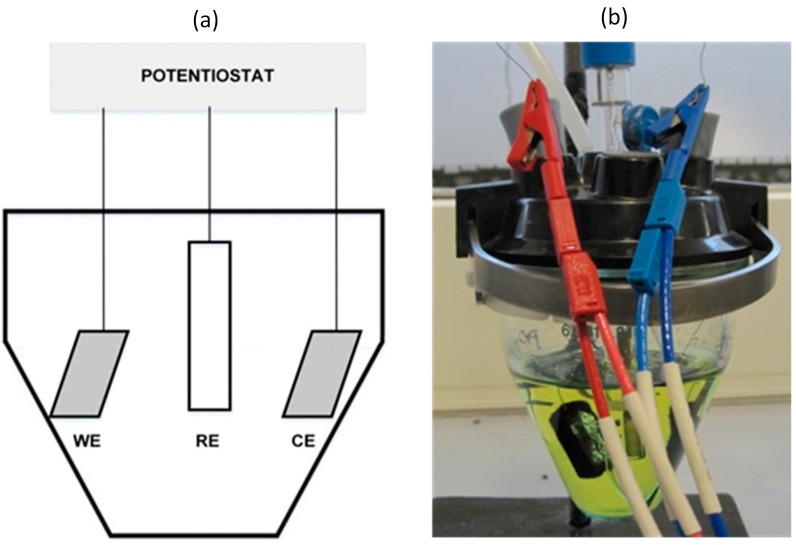
(**a**) Cell diagram (WE: working electrode; RE: reference electrode; CE: counter electrode) and (**b**) cell assembly.

The electrodes described in Section 2.4 were characterised in a 100 mL conical cell (Metrohm 6.1415.210), using a three-electrode configuration with an Ag/AgCl reference electrode (Bioblock Scientific), as shown in Figure 3. A solution containing 0.1 M KCl was used as the electrolyte and 3.4 mM K_3_Fe(CN)_6_ as the electroactive species. The reaction medium was previously sparged for 10 min with pure nitrogen to remove dissolved oxygen that interferes in the CV. The working and counter electrodes were identical in each test.

The analytical electrochemistry (CV, EIS, and CI; see supporting information, Figures S1–S3) was performed using a BioLogic VSP potentiostat (Biologic, Seyssinet-Pariset, France). The peak analysis was carried out using the software associated with the equipment (EC-Lab^®^ version 10.40, Biologic, Seyssinet-Pariset, France). The ohmic drop is compensated by an in-built method in the EC-Lab^®^ software in order to avoid undesirable peak displacement and current underestimates in the CV at relatively high currents.

## 3. Results and Discussion

In this section, we test and validate the methodology described in Section 2 on the materials shown in Table 1. Prior to its application, the ohmic drop was determined (Table 2) based on CI and EIS techniques repeated 10 times each (see supporting information, Section 1 and Section 2, Figures S1 and S2). As expected, samples made of the same material yielded lower resistance as the geometric surface area increases. This highlights the need of compensating for the ohmic drop in the CV.

### 3.1. Estimation of Electroactive Area

**Figure 4:**
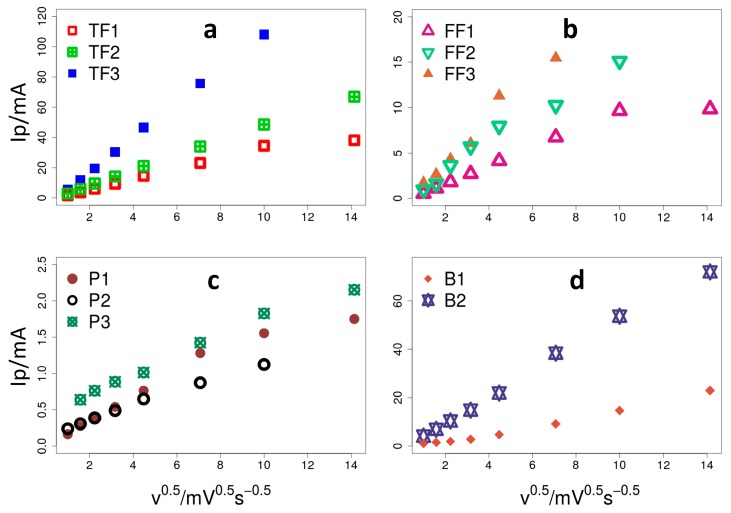
Randles-Ševčik plot (**a**) thick felt (TF); (**b**) fine felt (FF); (**c**) paper (P); (**d**) brush (B).

As discussed in Section 2, the first step in the proposed methodology is to determine the electroactive surface area, which is a critical parameter for the electrodes characterisation since it has a definite impact on the electrochemical reaction rate. Figure 4 illustrates the peak reduction currents (*I_p_*) versus the square root of the scan rate (*υ*^0.5^), which defines the Randles-Ševčik curves for the selected electrode material (see Section 2.3). As can be observed, for scan rates below 100 mV·s^−1^ (*υ*^0.5^ = 10) the trend is linear, being indicative of a semi-infinite diffusion regime. However, at scan rates above 100 mV·s^−1^ the current falls below the linear trend, which indicates the existence of irreversibilities [18]. Interestingly, this behaviour slightly differs for the carbon brush electrodes, where irreversibilities only appear at scan rates above 200 mV·s^−1^ (*υ*^0.5^ = 14.1), which seems to be indicative of enhanced electrode kinetics.

The EAs can be estimated from the slope of the Randles-Ševčik profiles as shown in Table 3. This table also provides the ratio between the evaluated EA and the apparent surface of the electrodes (AS), which normalises the EA to the electrode size.

Table 3 shows that for electrodes made of the same material, the EA/AS ratio is very similar regardless of the size of the electrode (see estimation of apparent surface area in supporting information), which also proves that the EA/AS ratio can be safely used to compare electrodes with different geometries.

Interestingly, carbon paper electrodes showed an EA/AS far below those observed on the other electrodes (See Table 3). This is indeed a noteworthy observation, for a rough carbonaceous material would be expected to display high EA/AS ratios (as with the other carbonaceous electrodes). An unexpected low EA/AS is explained by an unusually low EA, most probably due the presence of electrochemically inactive areas within the surface of the electrode. These inactive areas can be caused by partial fouling, chemical inactivation or catalyst poisoning among others. Therefore, when comparing different electrode materials, the EA/AS can be used to detect irregularities attributable to the electrode surface deficiencies. In our particular case, we attribute this relatively low EA/AS for the paper electrodes (at least in part) to the embedded air that could not be removed in the pre-treatment process.

### 3.2. Determination of the Fractal Dimension

**Figure 5:**
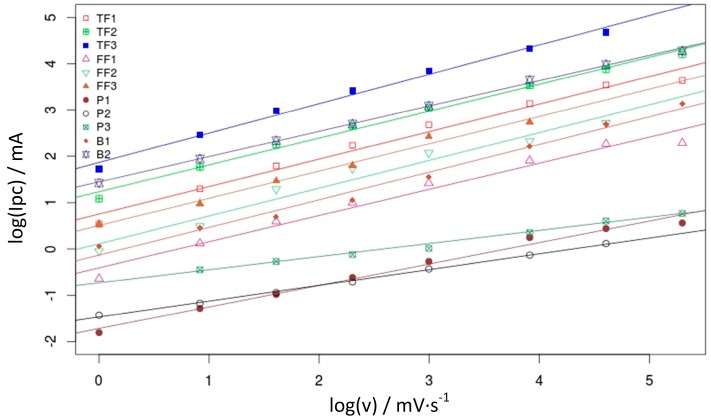
Linear trend in the logarithmic representation of *I_pc_* vs. *υ*.

This section deals with the evaluation of *D_f_*, a parameter that provides information about the relationship between the macroscopic and microscopic structure of an electrode, thus complementing the information provided by the EA. Following the procedure described in Section 2.3, *I_pc_* has been plotted against *υ* in Figure 5 on a logarithmic basis. This figure shows that all of the evaluated materials follow a linear model, indicating that *I_pc_* follows a power-law dependency versus the scan rate in the considered range. Fractal dimension can be calculated from this slope as described in Section 2.3. 

**Figure 6:**
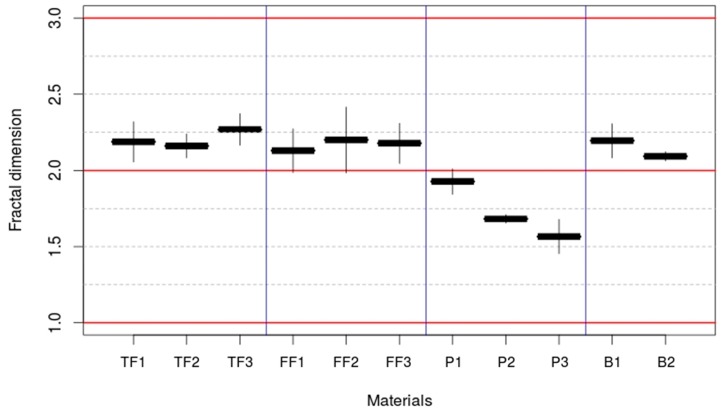
Fractal dimension of the electrodes tested. The values corresponding to Euclidean integer dimensions are shown as red solid lines.

The slope of the linear fits shown in Figure 5 corresponds to the parameter α in Equation (3). This is shown in Table 4 and allows to calculate *D_f_* (Figure 6) (See Section 2.3). 

The felt and brush electrodes showed a *D_f_* > 2, which indicates that the surfaces present an intrinsic three-dimensional structure. This feature is expected to be uniformly distributed in the electrode principal plane according to its scaling properties [26]. However, the *D_f_* found for carbon paper electrodes was even lower than the value expected for a completely smooth surface, which is *D_f_* = 2. This may be caused by the accumulation of gas bubbles on the surface of the electrodes that could not be removed in the pre-treatment process. This result corroborates the unexpected EA/AS observed for these electrodes (see Section 3.1). Interestingly, Figure 6 shows that as electrode size increases from P1 to P3, *D_f_* gets further reduced, which can be attributed to a higher proportion of air bubbles in larger size electrodes (air bubbles tend to accumulate in central region of the electrode and far from the edges). This fact enforces the information given by low EA/AS values about the presence of “irregularities/impurities” that inactivate certain sections of the electrodes.

**Figure 7:**
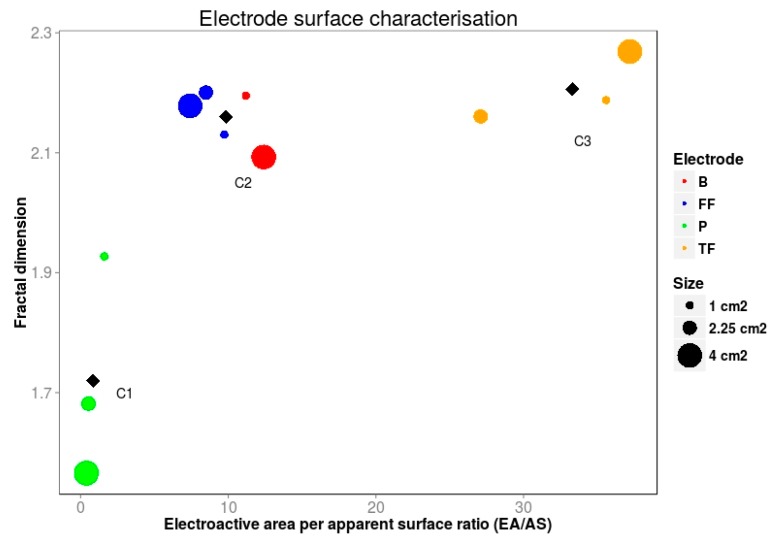
Relationship between fractal dimension and EA/AS for each material tested. Black diamond points show the centroids of electrode clusters.

The information provided by the *D_f_* can be integrated with the information provided by EA/AS, as shown in Figure 7. In our particular case, the different electrodes tested have been arranged in three different groups (classified via k-means algorithm with C1, C2 and C3, representing the centroids) for each electrode material and electrode size. An efficient electrode should have a high EA/AS and a fractal dimension greater than two and as close to three as possible. Therefore, this electrode should appear within the region dominated by C3. Moreover, a high fractal dimension points to the existence of a three-dimensional subjacent structure which often results in high EA/AS. Thus, it is unlikely to find materials below the main diagonal in Figure 7, which results in a forbidden area.

It is also interesting to point out that by graphically integrating the information provided by the electroactive area and the fractal dimension, we can easily uncover unexpected behaviours of the materials under examination. For example, materials with quite different morphologies such as FF and B can surprisingly display similar electrochemical performance as they fall within the same region (C2) in Figure 7. In contrast, materials with an a priori “enhanced” three-dimensional structure such as TF and B that would be expected to perform similarly, they actually fall within different regions in Figure 7. Although they show comparable fractal dimensions, their electroactive area differs significantly. Therefore, in this particular case, EA would become the key parameter in a potential screening process. 

Overall, the described approach combines graphically two estimators (EA/AS and *D_f_*) derived from a common experimental procedure that provides complementary information which is useful not only for quantifying the merit of electrode materials according to their reactive area, but also to highlight unexpected behaviours of the materials under test.

## 4. Conclusions

An experimental framework for comparing the surface properties and electrochemical efficiency of carbon electrodes, focused on BES development, is proposed. The usual evaluation of electrochemical active area alone does not provide sufficient information to estimate the performance of an electrode. Here we also calculate the fractal dimension to account for the 3D structure of the material. By combining the information provided by these two complementary parameters we can have an estimation of the behaviour of electrode materials. The results obtained during the validation of the method show its suitability at least to characterise and compare carbonaceous electrode materials, offering an alternative metric for surface evaluation. Moreover, by graphically integrating the information provided by the electroactive area and the fractal dimension, this method makes it easy to highlight unexpected behaviours of the materials under examination. Although it does not substitute accurate characterisation methods, it provides a suitable platform for easy comparison of a wide variety of different carbon-based materials, which are the most common electrode materials used in BES. For researchers and developers with limited budgets, it represents a cost-effective methodology since it can be performed using a standard potentiostat, which is present in most of bioelectrochemistry laboratories. Therefore, this method can become a useful tool for the screening and preliminary selection of available electrode materials during BES scale-up processes.

## Figures and Tables

**Table 1:** Material specification and coding.

Code	Material	Size	Apparent Surface (cm^2^)
TF1	Thick carbon felt	1 cm width; 1 cm length; 5 mm thickness	1
TF2	Thick carbon felt	1.5 cm width; 1.5 cm length; 5 mm thickness	2.25
TF3	Thick carbon felt	2 cm width; 2 cm length; 5 mm thickness	4
FF1	Fine carbon felt	1 cm width; 1 cm length; 2 mm thickness	1
FF2	Fine carbon felt	1.5 cm width; 1.5 cm length; 2 mm thickness	2.25
FF3	Fine carbon felt	2 cm width; 2 cm length; 2 mm thickness	4
P1	Carbon paper	1 cm width; 1 cm length	1
P2	Carbon paper	1.5 cm width; 1.5 cm length	2.25
P3	Carbon paper	2 cm width; 2 cm length	4
B1	Carbon brush	1 cm diameter; 2.5 cm height	1.87
B2	Carbon brush	2 cm diameter; 3 cm height	5.33

**Table 2:** Ohmic drop of each cell set-up. The standard error of the mean estimates the uncertainty associated to the determination of the ohmic drop.

Electrodes	Mean Ohmic Drop (Ω)	Standard Error
TF1	22.50	0.010
TF2	16.65	0.011
TF3	10.09	0.012
FF1	34.08	0.011
FF2	18.05	0.011
FF3	20.02	0.013
P1	18.29	0.011
P2	14.05	0.010
P3	9.81	0.012
B1	13.71	0.009
B2	8.16	0.010

**Table 3:** Electroactive areas.

Material	Slope	Electroactive Area (cm^2^)	Electroactive Area per Apparent Surface Area Ratio (EA/AS)
TF1	9.44 × 10^−2^	37.16	37.2
TF2	1.55 × 10^−1^	61.03	27.1
TF3	3.62 × 10^−1^	142.47	35.6
FF1	2.47 × 10^−2^	9.73	9.73
FF2	4.85 × 10^−2^	19.10	8.49
FF3	7.53 × 10^−2^	29.66	7.42
P1	4.07 × 10^−3^	1.60	1.60
P2	3.11 × 10^−3^	1.22	0.54
P3	3.91 × 10^−3^	1.54	0.39
B1	5.31 × 10^−2^	20.91	11.2
B2	1.68 × 10^−1^	65.97	12.4

**Table 4:** Fractal parameter comparison of different electrodes using CV measurements. The value presented alongside the fractal parameter α represents the 90% confidence intervals for each estimated parameter.

Electrode	Experimental Range *υ* (mV·s^−1^)	Fractal Parameter (α)	Correlation Coefficient (*R*^2^)
TF1	1–200	0.594 ± 0.066	0.981
TF2	1–200	0.580 ± 0.039	0.993
TF3	1–100	0.634 ± 0.052	0.992
FF1	1–200	0.565 ± 0.072	0.975
FF2	1–200	0.600 ± 0.108	0.961
FF3	1–50	0.589 ± 0.066	0.989
P1	1–200	0.464 ± 0.042	0.987
P2	1–100	0.341 ± 0.014	0.998
P3	2.5–200	0.283 ± 0.026	0.998
B1	1–200	0.598 ± 0.056	0.986
B2	1–200	0.547 ± 0.015	0.999

## Supplementary Materials

The following are available online at www.mdpi.com/1996-1944/10/1/79/s1.
