# Supplementary material for: Methodology for Fast and Facile Characterisation of Carbon-Based Electrodes Focused on Bioelectrochemical Systems Development and Scale Up
Source: Materials (Basel). 2017 Jan 20;10(1):79. doi: 10.3390/ma10010079 (PMC5344576; doi:10.3390/ma10010079)
Supplement: Supplementary file 1 [file materials-10-00079-s001.pdf]

Supplementary Materials: Methodology for Fast and Facile Characterisation of Carbon-Based Electrodes Focused on Bioelectrochemical Systems Development and Scale Up

Raúl Mateos, Raúl M. Alonso, Adrián Escapa and **Antonio Morán**

1. Ohmic Drop Determination

The current registered in the set of cyclic voltammetry experiments suggests the necessity to carry out ohmic drop compensation in order to obtain voltammograms suitable for data analysis [1].

The ohmic drop was determined using two different analytical techniques, current interrupt and electrochemical impedance spectroscopy; the final value used to compensate the cyclic voltammetry was the 85% of the average between both results—the mean value of 10 repetitions per technique.

The current interrupt technique consists in the measurement of ohmic drop by the application of Ohm's law once a known multi-step current signal is applied. The potential is measured immediately before and after the current has been interrupted, then the difference in the observed potentials is the ohmic drop. The ohmic drop divided by the known current, *I*, before the interrupt gives the ohmic resistance [2].

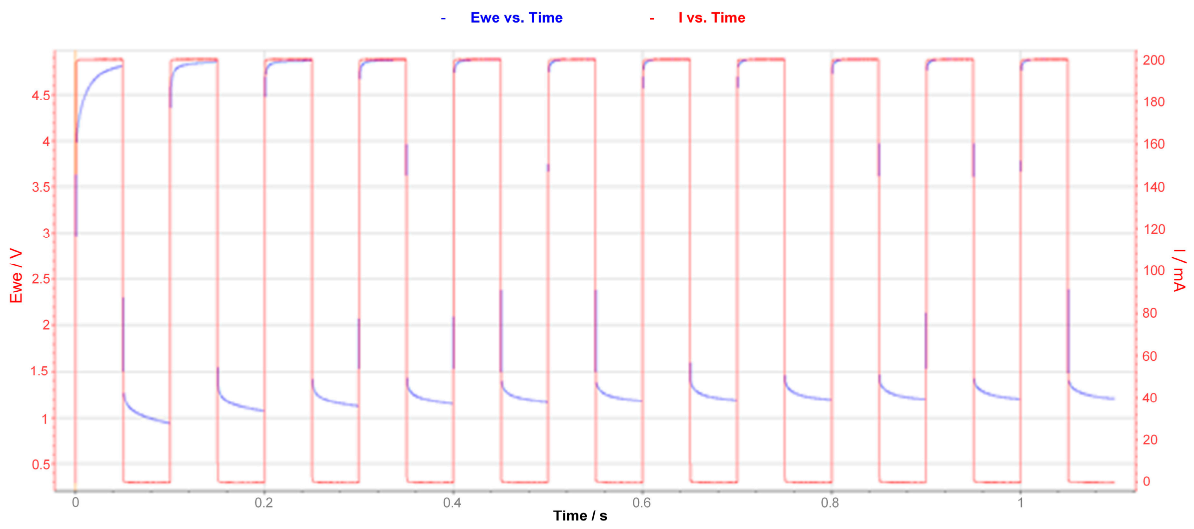

Figure S1. Example of current interrupt experiment performed in this study. (Ewe: Working electrode potential; *I*: Current).

Ohmic drop can also be determined by electrochemical impedance spectroscopy (EIS). This technique measures the impedance of a system over a range of frequencies by applying a variable frequency sinusoidal signal, superimposed to a fixed applied potential. In the Nyquist plot, the intersection of the impedance data with the real part of the axis at the high frequency end gives the ohmic resistance [2,3]. In our experimental procedure, the selected parameters are the following: Working electrode potential 0 V vs. open circuit potential; Perturbation amplitude 20 mV (root mean square value); Frequency range from 200 kHz to 100 mHz; 6 point per logarithmic decade; The first value of the EIS at high frequency is rejected to estimate EIS spectrum [3].

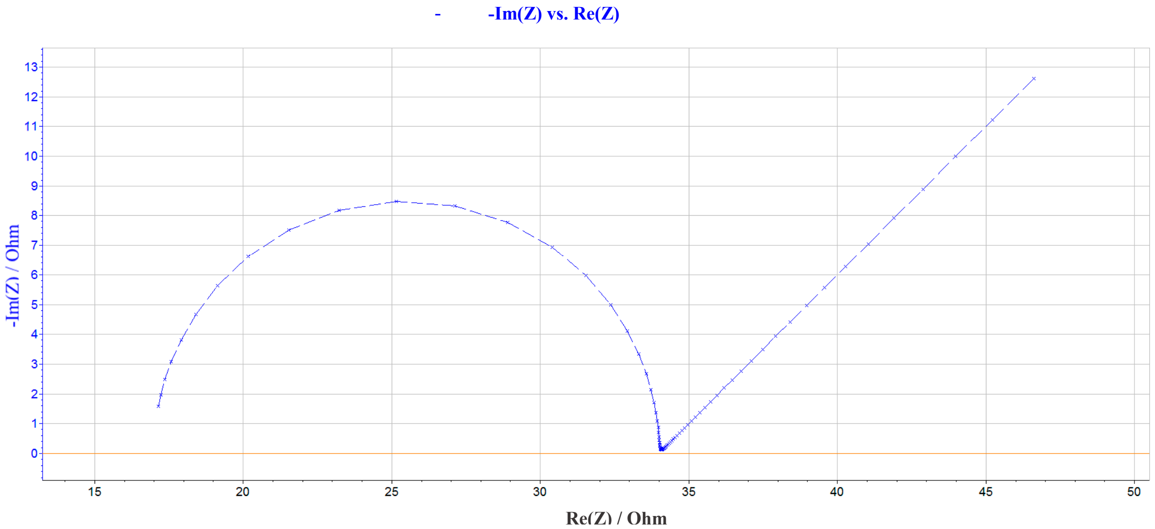

Figure S2. Model of a cell electrochemical impedance spectroscopy (EIS) experiment. (*Z*: Impedance).

2. Cyclic Voltammetry Experiments

Cyclic voltammetry experiments were performed in scan rates of 1, 2.5, 5, 10, 20, 50, 100 and 200 mV·s^{-1}. The highest scan rates (>50 mV·s^{-1}) showed a significant peak separation, which indicates process irreversibility due to sluggish electron transfer. The electrolyte was deaerated to avoid oxygen reaction artefacts in the CV experiments. Ohmic drop was compensated for in each test. Three examples of subsequent voltammetries on 1 cm^2 thick felt electrodes are shown below (Figure S3).

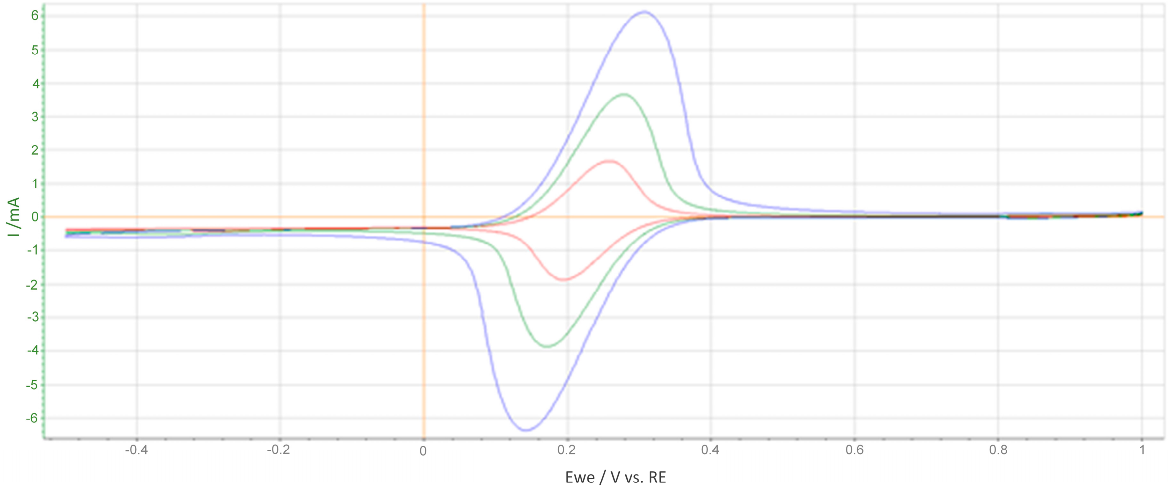

Figure S3. Example of voltammetries performed on 1 cm^2 thick felt.

3. Projected Brush Electrode Area Determination

The projected area of the brushes was calculated as a coverage percentage in order to compare the obtained electroactive area per apparent area (Table 3 in the paper). In the case of these electrodes the procedure consists in taking a digital image of the electrode over a white background. The image file is introduced in *R* data analysis software, where it is processed from an RGB model. The percentage of black pixels is then distinguished from the background using a threshold value for each channel. The coverage area is inferred from the percentage of black pixels obtained.

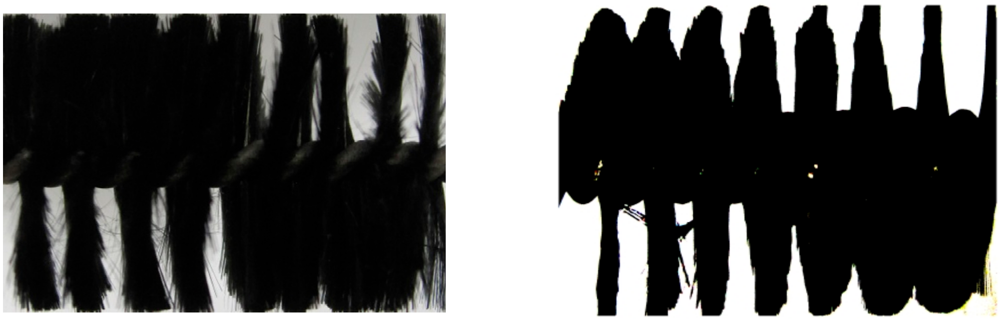

(**a**) (**b**)

Figure S4. Original brush image (**a**) and digitally treated brush image (**b**).

References

1. Zoski, C.G.C. *Handbook of Electrochemistry*; Elsevier: Amsterdam, The Netherlands, 2007.
2. Cooper, K.R.; Smith, M. Electrical test methods for on-line fuel cell ohmic resistance measurement. *J. Power Sources* **2006**, *160*, 1088–1095.
3. Orazem, M.E.; Tribollet, B. *Electrochemical Impedance Spectroscopy*; John Wiley & Sons: New York, NY, USA, 2011.
